# Ferroptosis, radiotherapy, and combination therapeutic strategies

**DOI:** 10.1007/s13238-021-00841-y

**Published:** 2021-04-23

**Authors:** Guang Lei, Chao Mao, Yuelong Yan, Li Zhuang, Boyi Gan

**Affiliations:** 1 Department of Radiation Oncology, Hunan Cancer Hospital and The Affiliated Cancer Hospital of Xiangya School of Medicine, Central South University, Changsha, 410013 China; 2 Department of Experimental Radiation Oncology, The University of Texas MD Anderson Cancer Center, Houston, TX 77030 USA; 3 The University of Texas MD Anderson UTHealth Graduate School of Biomedical Sciences, Houston, TX USA

**Keywords:** ferroptosis, lipid peroxidation, GPX4, SLC7A11, radiotherapy, immunotherapy, radiosensitization, combination therapy

## Abstract

Ferroptosis, an iron-dependent form of regulated cell death driven by peroxidative damages of polyunsaturated-fatty-acid-containing phospholipids in cellular membranes, has recently been revealed to play an important role in radiotherapy-induced cell death and tumor suppression, and to mediate the synergy between radiotherapy and immunotherapy. In this review, we summarize known as well as putative mechanisms underlying the crosstalk between radiotherapy and ferroptosis, discuss the interactions between ferroptosis and other forms of regulated cell death induced by radiotherapy, and explore combination therapeutic strategies targeting ferroptosis in radiotherapy and immunotherapy. This review will provide important frameworks for future investigations of ferroptosis in cancer therapy.

## INTRODUCTION

Regulated cell death (RCD), such as apoptosis, is a recognized hindrance to tumorigenesis. Consequently, cancer cells gradually evolve resistance to RCDs during tumor progression (Hanahan and Weinberg, 2011; Galluzzi et al., 2018; Green, 2019). Ferroptosis is a recently identified form of RCD driven by iron-dependent lipid peroxidation, which is distinct from other RCDs, such as apoptosis, autophagy and necroptosis, in morphology and mechanisms (Dixon et al., 2012; Stockwell et al., 2017). Inhibitors for these other RCDs generally are ineffective in blocking ferroptosis (Dixon et al., 2012) (although in some contexts ferroptosis is also considered a form of autophagy-dependent cell death (Gao et al., 2016; Hou et al., 2016)). Morphologically, ferroptosis is neither characterized by typical apoptotic features, such as chromatin condensation and apoptotic body formation, nor by the formation of autophagosomes, a key feature of autophagy; instead, ferroptotic cells generally exhibit shrunken mitochondria with increased mitochondrial membrane density and diminished mitochondrial cristae (Dixon et al., 2012; Stockwell et al., 2017). Mechanistically, polyunsaturated-fatty-acid-containing phospholipids (PUFA-PLs) in cellular membranes are susceptible for peroxidation under iron- and reactive oxygen species (ROS)-rich conditions. A toxic buildup of such lipid peroxides in cellular membranes eventually damages membrane integrity, leading to ferroptotic cell death (Stockwell et al., 2017).

Cells have evolved diverse array of ferroptosis defense systems, including glutathione peroxidase 4 (GPX4)-dependent and -independent systems, to detoxify lipid peroxides, thereby preventing their accumulation to lethal levels and maintaining cell survival (Stockwell et al., 2017; Zheng and Conrad, 2020). Accordingly, inactivation of such defense systems by genetic or pharmacological approaches provokes ferroptosis (Liang et al., 2019). Importantly, ferroptosis is not only associated with multiple pathologic conditions and diseases, but has also been identified as a natural barrier to cancer development. Inactivation of some tumor suppressors, such as tumor protein p53 (p53) and BRCA1 associated protein-1 (BAP1), promotes tumor development at least partly via suppressing tumor ferroptosis (Jiang et al., 2015; Zhang et al., 2018; Zhang et al., 2019b; Stockwell et al., 2020). Likewise, ferroptosis was recently shown to play an important role in some cancer therapies, and inducing tumor ferroptosis has emerged as a promising strategy for cancer treatment (Hassannia et al., 2019).

Radiotherapy (RT), a common cancer treatment modality, uses targeted delivery of ionizing radiation (IR) to eradicate cancer cells (Delaney et al., 2005; Jaffray, 2012). IR penetrates the tumor field and induces both direct and indirect cellular effects. It directly induces various types of DNA damage, such as base damage, single strand breaks (SSBs), and double strand breaks (DSBs) (Baidoo et al., 2013). In addition, IR elicits radiolysis of cellular water and stimulates oxidative enzymes to generate highly reactive OH ^•^ radicals as well as other ROS, including O_2_^•−^ and H_2_O_2_, which can subsequently attack nucleic acids, lipids, and proteins in a dose-dependent manner (Azzam et al., 2012; Reisz et al., 2014). These direct and indirect effects together trigger adverse cellular events in cancer cells, including cell cycle arrest, cellular senescence, and RCDs such as apoptosis; however, the potential role and mechanisms of other forms of RCD in RT remain to be further studied (Adjemian et al., 2020).

Recently studies revealed that IR induces potent ferroptosis and that ferroptosis represents an important part of RT-mediated anticancer effects (Lang et al., 2019; Lei et al., 2020; Ye et al., 2020). In clinic, RT generally needs to be combined with chemotherapy, targeted therapies, or immunotherapy to eliminate cancer cells. Notably, ferroptosis has also been linked to the efficacy of some of above-mentioned cancer therapies (Ma et al., 2016; Sun et al., 2016; Guo et al., 2018; Wang et al., 2019b). In the following sections, we first briefly review our current understanding of IR-induced signaling and cellular effects, as well as ferroptosis pathways and its inducers. We then discuss various aspects of IR-induced ferroptosis, including its known and other potential mechanisms, the role of ferroptosis modulators in radiosensitivity and RT-activated immune responses, potential interactions of ferroptosis with other IR-induced RCDs. Finally, we explore therapeutic implications of targeting ferroptosis in overcoming tumor radioresistance, the possibility of using ferroptosis regulators as potential predictive markers for RT efficacy, and the relevance of ferroptosis to RT combined with immunotherapy.

## IR-INDUCED SIGNALING AND CELLULAR EFFECTS

Once IR induces DNA damage, ataxia telangiectasia mutated (ATM) and ataxia telangiectasia and Rad3 related (ATR) serine/threonine kinases rapidly detect these damages and induce complex signaling cascades known as DNA damage response (DDR) that activate the downstream checkpoint kinases 1/2 (CHEK1/2), which then phosphorylate p53, among others, to arrest the cell cycle so that the damages in DNA can be corrected by DNA repair machineries (Huang and Zhou, 2020). The ultimate fate of these cells is at least partly determined by the severity of IR-induced DNA damage: if the damage can be fully repaired, cells survive and reenter into cell cycle; in contrast, irreparable or improperly repaired DNAs in the genome will trigger senescence (a permanent state of cell cycle arrest), apoptosis, or other forms of RCD, the exact outcome of which is often related to the radiation dose, linear energy transfer (LET), cell types, and the status of key cellular factors, including p53 (Maier et al., 2016).

Regarding p53’s function in RT, p53 is stabilized and activated by RT, and then operates as a transcription factor to govern the transcription of diverse genes such as *cyclin dependent kinase inhibitor 1A* (*CDKN1A*/*p21)*, *plasminogen activator inhibitor-1 (PAI-1)*, and *promyelocytic leukemia protein (PML)*, which function to permanently arrest the cell cycle, thereby contributing to senescence (Bieging et al., 2014). Senescence is the terminus of most irradiated normal cells and a barrier for cancer development (Braig et al., 2005; Maier et al., 2016). Since *p53* is frequently mutated in cancer cells, other senescence checkpoints, such as the p16-retinoblastoma (RB) pathway, are also responsible for eliminating cancer cells upon IR (Sabin and Anderson, 2011). Notably, some of the senescent cells may also eventually undergo apoptosis. It has been indicated that the more potent and prolonged the activation of p53 by IR is, the more likely cells will undergo apoptosis rather than senescence (Vousden, 2000; Mijit et al., 2020). To induce apoptosis, p53 activation upregulates the expression of genes such as *p53 upregulated modulator of apoptosis* (*PUMA*), *BCL2-Associated X* (*BAX*), and *phorbol-12-myristate-13-acetate-induced protein 1* (*NOXA*), leading to irreversible mitochondrial outer membrane permeability (MOMP), which releases cytochrome C and activates the caspase-9/3/7 pathway, thereby inducing intrinsic apoptosis (Aubrey et al., 2018); alternatively, p53 induces the death receptors FAS (CD95), death receptor 5 (DR5) and FAS ligands, ultimately activating caspase-8 and its downstream effectors to trigger extrinsic apoptosis (Sheikh and Fornace, 2000).

RT can also induce other apoptosis-independent RCDs. Specifically, IR has been shown to induce autophagy or necroptosis in certain contexts. There exists a complex interaction between IR and autophagy (a cellular process wherein intracellular cargos are degraded in autophagosomes and recycled into the cytosol) (Hu et al., 2016). Multiple factors, such as ATM, AMP-activated protein kinase (AMPK), Sirtuin 1 (SIRT-1), and mitochondrial ROS, contribute to the induction of autophagy by IR, and autophagy can exert a pro-survival or pro-cell death function in IR-mediated cellular effects, depending on the context (Bristol et al., 2012; Hu et al., 2016). Therefore, the exact role of autophagy in radiosensitization remains somewhat controversial. Necropotosis is a caspase-independent RCD triggered by the phosphorylation-dependent activation of mixed lineage kinase domain like pseudokinase (MLKL) mediated by the receptor-interacting serine/threonine protein kinases 1/3 (RIPK1/3) complex. Recent studies suggest that IR can induce necropotosis in certain cancer cells, although necroptosis appears not to be the predominant RCD in response to IR (Nehs et al., 2011; Adjemian et al., 2020).

In addition, although mitotic catastrophe, a mechanism of abnormal mitosis-induced cell death, is a common cellular effect of RT, it is not strictly considered as an RCD (Galluzzi et al., 2018). Cells in mitotic catastrophe are almost unable to replicate, and the vast majority of cells eventually die, with only a small fraction resuming proliferation (Vakifahmetoglu et al., 2008). Finally, necrosis, as a non-RCD triggered by IR, is more commonly associated with the side effects of RT, such as cerebral or pulmonary radiation necrosis (Song and Colaco, 2018; Benveniste et al., 2019). In brief summary, IR can induce complex downstream signaling networks and trigger a diverse array of adverse cellular effects.

## FERROPTOSIS PATHWAYS AND INDUCERS

**Figure 1:**
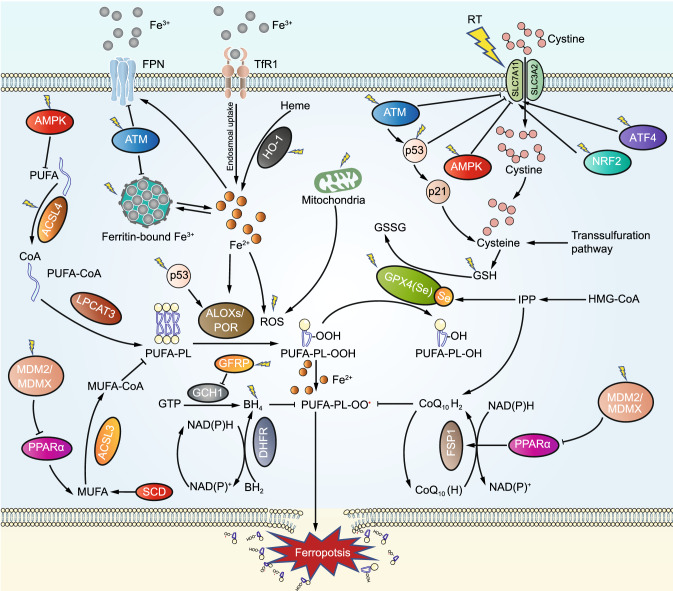
**The ferroptosis signaling pathway and ferroptosis regulators with known and potential relevance to radiotherapy.** Ferroptosis is driven by the accumulation of PUFA-PL peroxides, whose generation is facilitated by iron metabolism, PUFA-PL synthesis and peroxidation. Ferroptosis is counteracted by ferroptosis defense systems including the SLC7A11-GSH-GPX4, NAD(P)H-FSP1-CoQ, and GCH1-BH4 axes. Several regulators in the ferroptosis pathway that are modulated by radiotherapy are also highlighted. These regulators either have been confirmed to play roles or potentially might have roles in radiotherapy-induced ferroptosis

The accumulation of iron-dependent lipid peroxides is the cornerstone of ferroptosis (Dixon et al., 2012; Stockwell et al., 2017; Zheng and Conrad, 2020). Under normal conditions, ferroptosis defense systems can detoxify lipid peroxides and maintain them at non-toxic levels. When ferroptosis-executing systems override ferroptosis defense systems (such as when ferroptosis defense systems become largely defective), lipid peroxides quickly accumulate to toxic levels in cellular membranes, triggering ferroptosis (Stockwell et al., 2020; Zheng and Conrad, 2020) (Fig. 1). In this section, we discuss ferroptosis defense systems (including both GPX4-dependent and -independent systems) and ferroptosis-executing systems (including PUFA-PL metabolism and peroxidation, and iron metabolism). To facilitate our later discussion on targeting ferroptosis in overcoming radioresistance, we will also introduce ferroptosis inducers (FINs, the compounds capable of inducing ferroptosis in cancer cells) in this section. We refer readers to other excellent reviews for more extensive introduction of ferroptosis mechanisms (Stockwell et al., 2017; Stockwell et al., 2020; Zheng and Conrad, 2020).

### GPX4-dependent system

The solute carrier family 7 member 11-glutathione-GPX4 (SLC7A11-GSH-GPX4) signaling axis is believed to constitute the predominant ferroptosis defense system; indeed, ferroptosis was originally uncovered based on studies on this signaling axis (Dixon et al., 2012; Angeli et al., 2014; Dixon et al., 2014; Yang et al., 2014) (Fig. 1). SLC7A11 (also known as xCT) is a core component of the cystine/glutamate antiporter system x_c_^−^, and mediates the antiporter activity of system x_c_^−^ by importing extracellular cystine and exporting intracellular glutamate (Sato et al., 1999; Koppula et al., 2018). SLC7A11 takes up extracellular cystine and subsequently cystine is rapidly reduced to cysteine in cytosol through a nicotinamide adenine dinucleotide phosphate (NADPH)-consuming reduction reaction (Conrad and Sato, 2012; Koppula et al., 2018; Liu et al., 2020c; Liu et al., 2020d). Cysteine then serves as the rate-limiting precursor for the biosynthesis of GSH, a principle cofactor for GPX4 to detoxify lipid peroxides (Koppula et al., 2020). Blocking SLC7A11 transporter activity or depriving cystine in culture media induces potent ferroptosis in many cancer cells (Koppula et al., 2020). Notably, several tumor suppressors, including p53, BAP1, ADP-ribosylation factor (ARF), and Kelch-like ECH-associated protein 1 (KEAP1), promote ferroptosis by suppressing the expression or activity of SLC7A11 as part of their tumor suppressive activities (Jiang et al., 2015; Chen et al., 2017b; Fan et al., 2017; Zhang et al., 2018). Likewise, activating transcription factor 3 (ATF3) represses SLC7A11 expression by binding to the *SLC7A11* promoter, boosting the sensitivity of cancer cells to ferroptosis (Wang et al., 2020). SLC7A11 can be induced under various stress conditions, such as oxidative stress and amino acid starvation, by stress-responsive transcription factors such as nuclear factor erythroid 2-related factor 2 (NRF2) and activating transcription factor 4 (ATF4), thereby protecting cells from ferroptosis under stress conditions (Habib et al., 2015; Chen et al., 2017a; Fan et al., 2017). SLC7A11 can also be regulated at posttranscriptional levels. For example, the cancer stem cell marker CD44 and the deubiquitinating enzyme OTU domain-containing ubiquitin aldehyde binding protein 1 (OTUB1) promote ferroptosis resistance through stabilizing SLC7A11 (Ishimoto et al., 2011; Chew et al., 2017; Liu et al., 2019b).

It should be noted that in some cancer cells the transsulfuration pathway can supply a portion of intracellular cysteine for GSH synthesis through de novo synthesis of cysteine (Zhu et al., 2019), and enzymes that are involved in or regulated by the transsulfuration pathway (Fig. 1), such as cystathionine β-synthase (CBS) and cysteinyl-tRNA synthetase (CARS), can modulate the susceptibility of cancer cells to ferroptosis (Hayano et al., 2016; Wang et al., 2018). However, it is believed that intracellular cysteine derived from the transsulfuration pathway generally is not sufficient to cope with the high levels of oxidative stress to which cancer cells are exposed, and therefore most cancer cells still rely primarily on the acquisition of cysteine from the extracellular milieu via SLC7A11 (Chio and Tuveson, 2017; Koppula et al., 2020).

GPX4 utilizes GSH as its cofactor to reduce PL hydroperoxides to non-toxic PL alcohols, thereby maintaining the integrity of PL bilayers and preventing ferroptosis (Stockwell et al., 2017; Stockwell et al., 2020) (Fig. 1). GPX4 inactivation, pharmacologically or genetically, leads to drastic accumulation of toxic lipid peroxides and triggers ferroptosis (Angeli et al., 2014; Yang et al., 2014). Regarding GPX4’s role in cancer, GPX4 is overexpressed in a variety of cancers, and *Gpx4*+/− mice exhibit delayed lymphomagenesis compared with their wild-type counterparts (Ran et al., 2007; Zhang et al., 2020). Certain cancer cells, such as drug-tolerant persister cancer cells or therapy-resistant high-mesenchymal ones, are highly dependent on GPX4 activity, thereby exposing potential vulnerabilities for therapeutic targeting (Ran et al., 2007; Hangauer et al., 2017; Viswanathan et al., 2017). GPX4 is a selenoprotein; the selenocysteine (Sec) residue in GPX4 is required for its anti-ferroptosis activity (Angeli and Conrad, 2018; Ingold et al., 2018). Selenium supplementation not only promotes GPX4 protein synthesis, but also drives its transcription, while perturbation of the mevalonate pathway impairs translation of selenoproteins (including GPX4), thereby sensitizing cells to ferroptosis (Ingold et al., 2018; Alim et al., 2019; Conrad and Proneth, 2020). Together, SLC7A11-mediated cystine uptake, GSH biosynthesis, and GPX4 activity constitute a robust ferroptosis defense system that keeps lipid hydroperoxides at levels below the toxic threshold to maintain cell survival.

### GPX4-independent defense systems

The NAD(P)H-ferroptosis suppressor protein 1-ubiquinone [NAD(P)H-FSP1-CoQ] signaling axis is a recently established ferroptosis defense system that operates in parallel to the SLC7A11-GSH-GPX4 axis (Bersuker et al., 2019; Doll et al., 2019) (Fig. 1). Derived from the mevalonate pathway and mainly synthesized in mitochondria, CoQ is not only an important element of the mitochondrial electron transport chain (ETC), but its reduced form, ubiquinol (CoQH_2_), also acts as a potent lipophilic antioxidant (Frei et al., 1990; Duberley et al., 2014; Shimada et al., 2016). FSP1, also known as apoptosis-inducing factor-associated mitochondrial-associated protein 2 (AIFM2), was previously suggested to participate in inducing apoptosis (Wu et al., 2002), but its role in apoptosis is complicated and somewhat controversial (Vařecha et al., 2007; Yang et al., 2011; Kaku et al., 2015; Nguyen et al., 2020). FSP1 functions as an oxidoreductase of CoQ (Marshall et al., 2005; Elguindy and Nakamaru-Ogiso, 2015). Recent studies revealed that FSP1 localizes on the plasma membrane and reduces CoQ to CoQH_2_ by consuming NAD(P)H, and CoQH_2_ subsequently inhibits ferroptosis by trapping lipophilic free radicals; consequently, the blockade of CoQ biosynthesis pathway abolishes FSP1’s ability to suppress ferroptosis (Bersuker et al., 2019; Doll et al., 2019). Importantly, the NAD(P)H-FSP1-CoQ axis acts as an independent system in concert with the SLC7A11-GSH-GPX4 system to protect cells from ferroptosis. FSP1 has been considered as a p53-responsive gene (Horikoshi et al., 1999; Ohiro et al., 2002; Wu et al., 2004); however, recent studies demonstrated that FSP1 expression is not affected by p53 activator nutlin-3 or doxorubicin (Bersuker et al., 2019; Doll et al., 2019). Furthermore, a few studies have linked the regulation of FSP1 to cAMP-response-element-binding protein (CREB) (Nguyen et al., 2020) or mouse double minute 2 homolog/murine double minute X (MDM2/MDMX) complex (Venkatesh et al., 2020), although the exact relevance and the biological contexts of these regulations to ferroptosis remain to be further investigated.

Finally, tetrahydrobiopterin (BH_4_) and its rate-limiting enzyme guanosine triphosphate cyclohydrolase 1 (GCH1) were recently identified as an alternative ferroptosis defense system independent of GPX4 (Kraft et al., 2019; Soula et al., 2020) (Fig. 1). BH_4_ is a robust radical-trapping antioxidant in cellular membranes and is capable of promoting the regeneration of CoQH_2_ and α-tocopherol to counteract lipid peroxidation and ferroptosis (Crabtree et al., 2009; Kraft et al., 2019; Soula et al., 2020). BH_4_ is regenerated from its oxidized form boron dihydride (BH_2_) via dihydrofolate reductase (DHFR); consequently, inactivation of DHFR significantly increases cellular vulnerability to ferroptosis (Soula et al., 2020).

### PUFA-PL synthesis and peroxidation

Free PUFAs, such as arachidonic acids (AAs) and adrenic acids (AdAs), are catalyzed mainly by acyl coenzyme A synthetase long chain family member 4 (ACSL4) to produce their acyl coenzyme A (CoA) derivatives (such as AA/AdA-CoA). Subsequently, these PUFA-CoAs are processed to form lysophospholipids (LysoPLs) and further incorporated into PLs (such as AA-PE and AdA-PE) by lysophosphatidylcholine acyltransferase 3 (LPCAT3) and other enzymes (Fig. 1). Correspondingly, ablation of ACSL4 or LPCAT3 suppresses PUFA-PL synthesis and dramatically promotes ferroptosis resistance (Dixon et al., 2015; Doll et al., 2017; Kagan et al., 2017). In addition, energy stress (a metabolic stress condition with ATP depletion) activates AMPK, which suppresses acetyl-CoA carboxylase (ACC, which converts acetyl-CoA to malonyl-CoA) and reduces PUFA-PL levels (likely because malonyl-CoA is required for AA or AdA synthesis), resulting in ferroptosis blockade (Lee et al., 2020; Li et al., 2020) (Fig. 1).

Due to the presence of bis-allylic moieties in PUFAs, PUFA-PLs are particularly vulnerable to peroxidation (Conrad and Pratt, 2019). Lipid peroxidation is believed to occur through both enzyme-mediated reactions and enzymatic independent reactions known as autoxidation, wherein lipid peroxides can be generated through free radical chain reactions which require iron and oxygen (Conrad and Pratt, 2019). Regarding the enzymes that drive lipid peroxidation, while lipid peroxidation was initially proposed to be mediated by lipoxygenases (ALOXs) (Yang et al., 2016), the role of ALOXs in lipid peroxidation was subsequently challenged (Shah et al., 2018), and more recent studies revealed that, at least in most cancer cell lines, cytochrome P450 oxidoreductase (POR) appears to play a more dominant role in mediating lipid peroxidation (Yan et al., 2020; Zou et al., 2020b) (Fig. 1).

Other types of PLs are also involved in ferroptosis regulation. Recently PUFA-containing ether PLs (PUFA-ePLs) were found to act as an alternative substrate for lipid peroxidation (Zou et al., 2020a). In addition, supplementation of certain exogenous monounsaturated fatty acids (MUFAs) can displace PUFAs from PLs located in cellular membranes and render cells less susceptible to peroxidation, thereby attenuating ferroptosis (Magtanong et al., 2019). MUFA biosynthesis is mediated by stearoyl coenzyme A desaturase (SCD), and its incorporation into PLs requires acyl coenzyme A synthetase long chain family member 3 (ACSL3); correspondingly, SCD and ACSL3 have been shown to protect cells against ferroptosis (Paton and Ntambi, 2009; Magtanong et al., 2019; Tesfay et al., 2019) (Fig. 1).

### Iron metabolism

The labile iron generates free radicals and mediates lipid peroxidation through Fenton reaction (Ayala et al., 2014). Iron chelation by desferoxamine (DFO) blocks ferroptosis (therefore its name “ferroptosis”), whereas increases in labile iron levels sensitizes cells to ferroptosis, establishing that iron is fundamental to ferroptosis (Dixon et al., 2012; Kim et al., 2016). Labile iron pool is primarily maintained by proteins responsible for its uptake, storage, and export (Fig. 1). Iron uptake relies primarily on transferrin receptor 1 (TFR1), which transports ferritin-bound iron into cells via receptor-mediated endocytosis; notably, TFR1 was also recently identified as a biomarker for ferroptosis (Anderson and Vulpe, 2009; Gao et al., 2015; Feng et al., 2020). Iron is principally stored in ferritin in the form of Fe (III) (inert iron), which is not involved in lipid peroxidation; therefore, the abundance of ferritin, especially ferritin heavy chain (FTH1), is critical for ferroptosis suppression (Mumbauer et al., 2019). Ferritinophagy, the autophagic degradation of ferritin, promotes the release of iron stored in ferritin into the labile iron pool, thereby sensitizing cells to ferroptosis (Gao et al., 2016). Iron is mainly exported by ferroportin 1 (FPN1), and iron export is further facilitated by prominin2, which regulates the formation of ferritin-containing multivesicular bodies and exosomes; correspondingly, inhibition of these proteins drives ferroptosis (Geng et al., 2018; Brown et al., 2019). Moreover, several enzymes essential for lipid peroxidation, such as ALOXs and POR, are iron-dependent, and Fe (II) that is not bound to these enzymes further accelerates the propagation of peroxides during lipid peroxidation, leading to extensive ferroptosis (Wenzel et al., 2017; Shah et al., 2018; Zou et al., 2020b) (Fig. 1).

### Ferroptosis inducers

Several classes of FINs have been identified and developed, including class I FINs that inhibit SLC7A11 activity and deplete GSH, class II FINs that directly inhibit GPX4 activity by covalently binding to selenocysteine at the active site of GPX4, class III FINs that activate squalene synthase (SQS), thereby indirectly depleting both CoQ and GPX4, as well as other types of FINs (Hassannia et al., 2019). Besides, various nanomaterials have been exploited to induce ferroptosis locally (Liang et al., 2019). These FINs not only provide valuable tools for ferroptosis studies, but also can be employed as potential therapeutic agents for cancer therapy. The detailed mechanisms of action and applications of these FINs are shown in Table 1.

**Table 1:** The identified FINs and ferroptosis promoters

Classification	Compound	Mechanism	*In vivo*	Clinic	Radiosensitizer
Class I FINs	Erastin	Inhibit SLC7A11 activity			√
	PE	Inhibit SLC7A11 activity	√		
	IKE	Inhibit SLC7A11 activity	√		√
	SAS	Inhibit SLC7A11 activity	√	√	√
	Sorafenib	Inhibit SLC7A11 activity	√	√	√
	Cyst(e)inase	Cyst(e)ine depletion	√		√
	Glutamate	Inhibit SLC7A11 activity			
	BSO	GSH depletion	√		√
	DPI2	GSH depletion			
	Cisplatin	GSH depletion	√	√	√
Class II FINs	1S,3R-RSL3	Inhibit GPX4 activity			√
	ML162	Inhibit GPX4 activity			√
	ML210	Inhibit GPX4 activity			
	Altretamine	Inhibit GPX4 activity	√	√	
	Withaferin A	Inactivate/deplete GPX4	√		√
Class III FINs	FIN56	Degrade GPX4, activate SQS and deplete CoQ_10_			√
	Statins (fluvastatin, simvas-tatin, lovastatin acid)	Inhibit HMG-CoA reductase (inhibit CoQ_10_ synthesis, reduce GPX4 expression)	√	√	√
Class IV FINs	Ferric ammonium citrate/sulfate	Iron loading			
	FeCl_2_	Iron loading	√		
	Hemoglobin	Iron loading	√	√	√
	Hemin	Iron loading	√	√	
	Nonthermal plasma	Promote the release of Fe^2+^ from ferritin	√		√
	Lapatinib + siramesine	Upregulate TfR1 and downregulate FPN1	√	√	√
	Salinomycin	Inhibit iron translocation and deplete ferritin	√	√	√
	Artesunate, DHA	Endogenous Fe^2+^ causes the cleavage of endoperoxide bridge	√	√	√
	FINO2	Inhibit GPX4 activity, Oxidize ferrous iron and lipidome			
Other FINs/promoters	BAY 87–2243	Inhibit mitochondrial complex I			√
	BAY 11–7085	Upregulate HMOX1			√
	Auranofin/Ferroptocide	Inhibit thioredoxin	√	√	√
	iFSP1	Inhibit FSP1			
	4-CBA	CoQ_10_ depletion	√		
	DAHP	Inhibit GCH1	√		
	Methotrexate	Inhibit DHFR	√	√	
	MF-438/ CAY10566	Inhibit SCD1	√		
	JQ-1	Promote ferritinophagy	√		√
Nanoparticles	AMSNs	GSH depletion	√		
	LDL‐DHA	Loading natural omega 3 fatty acid	√		
	ZVI NPs	Iron loading	√		
	FeGd-HN@Pt@LF/ RGD2	Increase intracellular Fe^2+^ and H_2_O_2_ levels	√		
	DGU:Fe/Dox	delivery system releasing Fe^3+^ and doxorubicin	√		
	SRF@Fe^III^TA	Consists of Fe^3+^ ion, tannic acid and sorafenib	√		
	PSAF NCs	Increase intracellular Fe^2+^ levels	√		
	MON‐p53	Iron loading, inhibit SLC7A11	√		

This table lists compounds currently known to induce or promote ferroptosis, including their classification, mechanism, suitability for *in vivo* administration, and availability as radiosensitizers

## FERROPTOSIS AND RT

Excessive ROS generated by RT through radiolysis of cellular water can damage biomolecules, including lipids, and therefore can be potentially linked to lipid peroxidation and ferroptosis. Previous studies suggested that IR can generate hydroxyl radicals and promote lipid peroxidation in lipid bilayers (Walden and Hughes; Shadyro et al., 2002). It was recently established by us and others that RT can trigger potent ferroptosis and that ferroptosis represents a critical part of RT-mediated tumor suppression (Lang et al., 2019; Lei et al., 2020; Ye et al., 2020). In this section, we summarize these recent findings on RT-induced ferroptosis, explore other potential mechanisms linking RT to ferroptosis, and further discuss the crosstalk between ferroptosis and other RT-induced cellular effects.

### The role and known mechanisms of RT-induced ferroptosis

**Figure 2:**
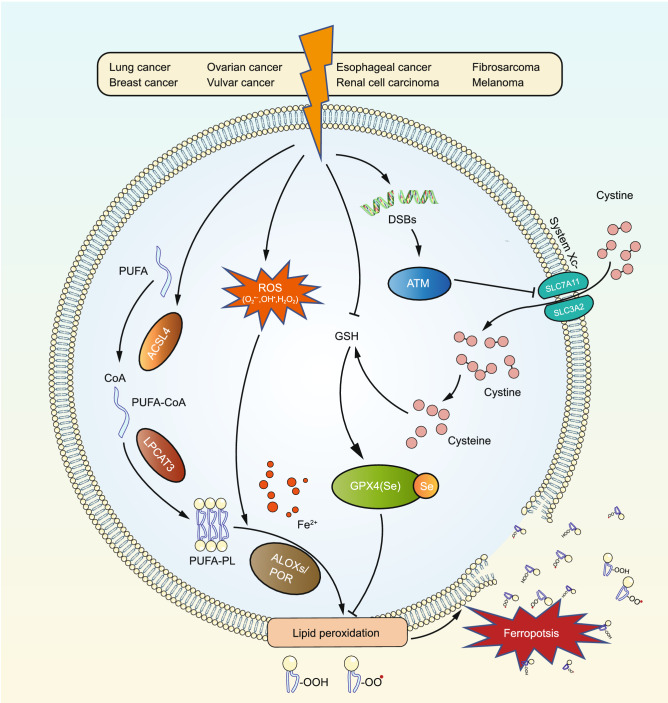
**Mechanisms of radiotherapy-induced ferroptosis.** Radiotherapy (RT) has been revealed to induce ferroptosis in the indicated cancers through several parallel pathways. RT-induced ROS in concert with RT-induced ACSL4 expression trigger PUFA-PL peroxidation and ferroptosis. RT also depletes GSH, dampens GPX4-mediated ferroptosis defense, thereby promoting ferroptosis. In addition, RT can repress SLC7A11 expression in an ATM-dependent manner to further promote ferroptosis or upregulate SLC7A11 expression as an adaptive response for ferroptosis protection, depending on the context

Substantial genetic and biochemical evidence forges a tight link between RT and ferroptosis in several cancers, including lung cancer, breast cancer, esophageal cancer, renal cell carcinoma, ovarian cancer, vulvar cancer, fibrosarcoma, and melanoma (Lang et al., 2019; Lei et al., 2020; Ye et al., 2020) (Fig. 2). First, RT is capable of significantly increasing the staining of C11-BODIPY and lipid peroxidation markers malondialdehyde (MDA) and 4-hydroxynonenal (4-HNE) in cancer cells and tumor samples, indicating that RT induces lipid peroxidation. Likewise, irradiated cells exhibit the increased expression of ferroptosis marker gene *prostaglandin-endoperoxide synthase 2* (*PTGS2*), as well as the morphologic feature of ferroptosis with shrunken mitochondria with enhanced membrane density. Ferroptosis inhibitors (ferrostatin-1 and liproxstatin-1) or iron chelator DFO can partially restore clonogenic cell survival following RT in a wide range of cancer cell lines; notably, the survival restoring effect by ferroptosis inhibitors is comparable to or even more pronounced than that by inhibitors of other RCDs such as apoptosis and necroptosis. To minimize damages to normal tissues, a high dose of IR is usually delivered through multiple low doses, which is called dose fractionation. Fractionation generally includes conventionally fractionation (such as 2 Gy once daily, 5 times/week), hypofractionation (such as 3 Gy once daily, 5 times/week), and hyperfractionation (such as 1.1 Gy twice daily, 5 times/week) (Withers, 1985; Williams et al., 2006). Notably, different RT doses and fractionation schedules result in differential levels of ferroptosis; specifically, lipid peroxidation and ferroptosis can be augmented with increasing doses in the single-fraction case, whereas the single fraction at 10 Gy induces more lipid peroxidation than three fractions with each fraction at 5 Gy (3 × 5 Gy) (Lang et al., 2019), which could provide insights for further investigations of ferroptosis in hypofractionated (e.g., stereotactic body radiation therapy), conventionally fractionated, and hyperfractionated RT (Thariat et al., 2013).

Mechanistically, IR induces lipid peroxidation and ferroptosis likely through at least three parallel pathways (Lang et al., 2019; Lei et al., 2020; Ye et al., 2020) (Fig. 2). First, IR can induce lipid peroxidation through generating excessive ROS. Specifically, IR-generated ROS can abstract electrons from PUFAs, resulting in the formation of PUFA radicals (PUFA^•^). Subsequently, these unstable carbon-centered radicals can interact promptly with oxygen molecules to produce lipid peroxyl radicals (PUFA-OO^•^), which then abstract H^•^ from other molecules via the Fenton reaction and eventually generate lipid hydroperoxides (PUFA-OOH) (Shadyro et al., 2002; Azzam et al., 2012). In addition, IR upregulates ACSL4 expression to promote PUFA-PLs biosynthesis, although the exact underlying mechanism by which IR induces ACSL4 levels remains unclear (Lei et al., 2020). Consistently, IR exerts a pronounced effect on ferroptosis-associated lipid metabolism, with multiple LysoPLs and diacylglycerols (DAGs) reported to be significantly increased following irradiation (Ye et al., 2020). Increased levels of LysoPLs (Colles and Chisolm, 2000; Dixon et al., 2015; Yang et al., 2016; Kagan et al., 2017; Zhang et al., 2019a) and DAGs (Zhang et al., 2019a; Zou et al., 2019) have also been observed following the treatment with FINs, suggesting that IR and FIN treatment induce similar lipidomic signatures, which is in line with their shared effects to trigger ferroptosis. Finally, IR also leads to GSH depletion, which weakens GPX4-mediated ferroptosis defense and further promotes ferroptosis (Ye et al., 2020).

In one study, IR was shown to repress SLC7A11 expression in an ATM-dependent manner, and it was proposed that IR-mediated SLC7A11 repression triggers ferroptosis by reducing cystine uptake and GSH synthesis (Lang et al., 2019) (Fig. 2). However, other studies revealed that the expression of SLC7A11 is actually induced by IR, likely as an adaptive response (Xie et al., 2011; Lei et al., 2020). Although the mechanism underlying the upregulation of SLC7A11 upon IR remains undefined, it likely involves NRF2 and/or ATF4, both of which are generally activated by IR and are known to regulate *SLC7A11* transcription (McDonald et al., 2010; Zong et al., 2017; Koppula et al., 2020). Therefore, it appears that IR can either activate or repress SLC7A11 expression in a context (cell line, IR dose or duration)-dependent manner. Taken together, the multifaceted evidence from different studies establishes a robust link between ferroptosis and RT, and suggests several underlying mechanisms for RT-induced ferroptosis.

### Other potential mechanisms

Because multiple metabolic pathways are involved in the regulation of ferroptosis and several ferroptosis regulators are RT-responsive genes (Fig. 1), other potential mechanisms might also contribute to RT-induced ferroptosis, which will be further discussed in this subsection. As a central effector of RT, p53 is not only activated by IR (Fei and El-Deiry, 2003; Gudkov and Komarova, 2003), but also plays a dual role in the ferroptosis network (Kang et al., 2019) (Fig. 1). Specifically, p53 was shown to repress *SLC7A11* transcription by binding directly to the p53 response element in the *SLC7A11* promoter region or by interacting with ubiquitin-specific protease 7 (USP7) to reduce the levels of H2B monoubiquitination on the *SLC7A11* gene regulatory region, thereby exerting a pro-ferroptosis effect in response to oxidative stress (Jiang et al., 2015; Wang et al., 2019c). It was further shown that p53-mediated SLC7A11 repression promotes ferroptosis in an ALOX12-dependent manner (Chu et al., 2019). In addition, p53 can induce the expression of spermidine/spermine N1-acetyltransferase 1 (SAT1) to upregulate ALOX15, thus promoting ferroptosis upon ROS stress (Ou et al., 2016). p53 regulation of glutaminases 2 (GLS2) (Hu et al., 2010; Suzuki et al., 2010; Gao et al., 2015) or ferredoxin reductase (FDXR) (Hwang et al., 2001; Zhang et al., 2017) might also potentially contribute to ferroptosis. In contrast, other studies showed that p53 can function as a ferroptosis inhibitor by upregulating p21 to maintain the GSH levels upon metabolic stress (Tarangelo et al., 2018), or by blocking dipeptidyl-peptidase-4 (DPP4) activity in a transcription-independent manner (Xie et al., 2017). Given the context dependent role of p53 in governing ferroptosis, whether RT-induced p53 activation contributes to or antagonizes RT-induced ferroptosis merits further investigations.

Another signaling node that potentially links RT to ferroptosis is AMPK. RT has been widely demonstrated to activate AMPK (Sanli et al., 2010; Sanli et al., 2014). Interestingly, AMPK activation also appears to exert context dependent effects on ferroptosis (Fig. 1). AMPK-mediated phosphorylation of beclin-1 was reported to inhibit system x_c_^−^ activity, thereby promoting ferroptosis (Song et al., 2018), while energy stress-induced AMPK activation was recently shown to inhibit ferroptosis by restraining PUFA-PL biosynthesis (Lee et al., 2020; Li et al., 2020). The exact role of AMPK in RT-induced ferroptosis therefore remains to be examined.

It is known that RT induces the expression of MDM2 in an ATM- or p53-dependent manner (Chen et al., 1994; Maya et al., 2001). Recently MDM2 was shown to promote ferroptosis through regulating lipid metabolism and FSP1 expression (Venkatesh et al., 2020), suggesting a possible role of MDM2 in RT-induced ferroptosis (Fig. 1). As discussed in a previous section, The GCH1-BH_4_ signaling axis constitutes a GPX4-independent ferroptosis defense system. It was observed that IR decreased the level and bioavailability of BH_4_ *in vivo*, presumably because IR induces the expression of GCH1 feedback regulatory protein (GFRP), thereby potentiating GFRP-mediated inhibition of GCH1 activity (Li et al., 2010; Berbee et al., 2011; Cheema et al., 2014; Pathak et al., 2014) (Fig. 1). This raises the possibility that GCH1 might also be involved in regulating IR-induced ferroptosis. Furthermore, IR promotes iron release from heme by inducing heme oxygenase-1 (HO-1) or from ferritin (Han et al., 2005; Wolszczak and Gajda, 2010; Hassannia et al., 2018) (Fig. 1). However, it has also been reported that IR upregulates the expression of FTH1 (Choudhary et al., 2020), which plays an important role in reducing oxidative stress and promoting radioresistance (Pang et al., 2016). Therefore, it remains obscure whether RT promotes ferroptosis by regulating iron metabolism.

Finally, transmission electron microscopy revealed that mitochondria exhibit ferroptotic cell features following IR, implying that mitochondria are potentially involved in IR-induced ferroptosis (Lei et al., 2020). Indeed, IR has been shown to dramatically alter mitochondrial structure or function, including mitochondrial DNA, mitochondrial permeability, ETC activity, oxidative phosphorylation, and mitochondrial antioxidant enzyme function, resulting in extensive mitochondrial ROS production (Leach et al., 2001; Kam and Banati, 2013) (Fig. 1). Further studies are required to determine the potential role of mitochondria in IR-induced ferroptosis.

Collectively, diverse signaling nodes and cellular processes have been linked to both RT and ferroptosis; therefore, RT-induced ferroptosis likely involve a multitude of mechanisms. Future investigations are needed to develop a comprehensive molecular understanding of RT-induced ferroptosis.

### Crosstalk between ferroptosis and other RT-induced cellular effects

As introduced in a preceding section, one major cellular effect triggered by RT is to induce DNA damage in the nucleus. Ferroptosis, on the other hand, is triggered by lipid damage, namely lipid peroxidation, caused by toxic accumulation of lipid hydroperoxides on cellular membranes. This raised the question of whether there exists any interaction between ferroptosis and DNA damage upon RT. Recent studies revealed that neither perturbation of IR-induced ferroptosis by ferroptosis inhibitors nor augmentation of IR-induced ferroptosis by FINs affects IR-mediated DSBs (Lei et al., 2020; Ye et al., 2020). Further, microbeam radiation analysis showed that IR specifically targeting the nucleus induced phosphorylated H2A histone family member X (γH2AX, a DSB marker) but did not produce 4-HNE (a lipid peroxidation marker), whereas levels of 4-HNE, but not those of γH2AX, were elevated following IR specifically targeting the cytoplasm, suggesting that ferroptosis induction in the cytoplasm and DNA damage in the nucleus can be uncoupled following IR (Ye et al., 2020).

**Figure 3:**
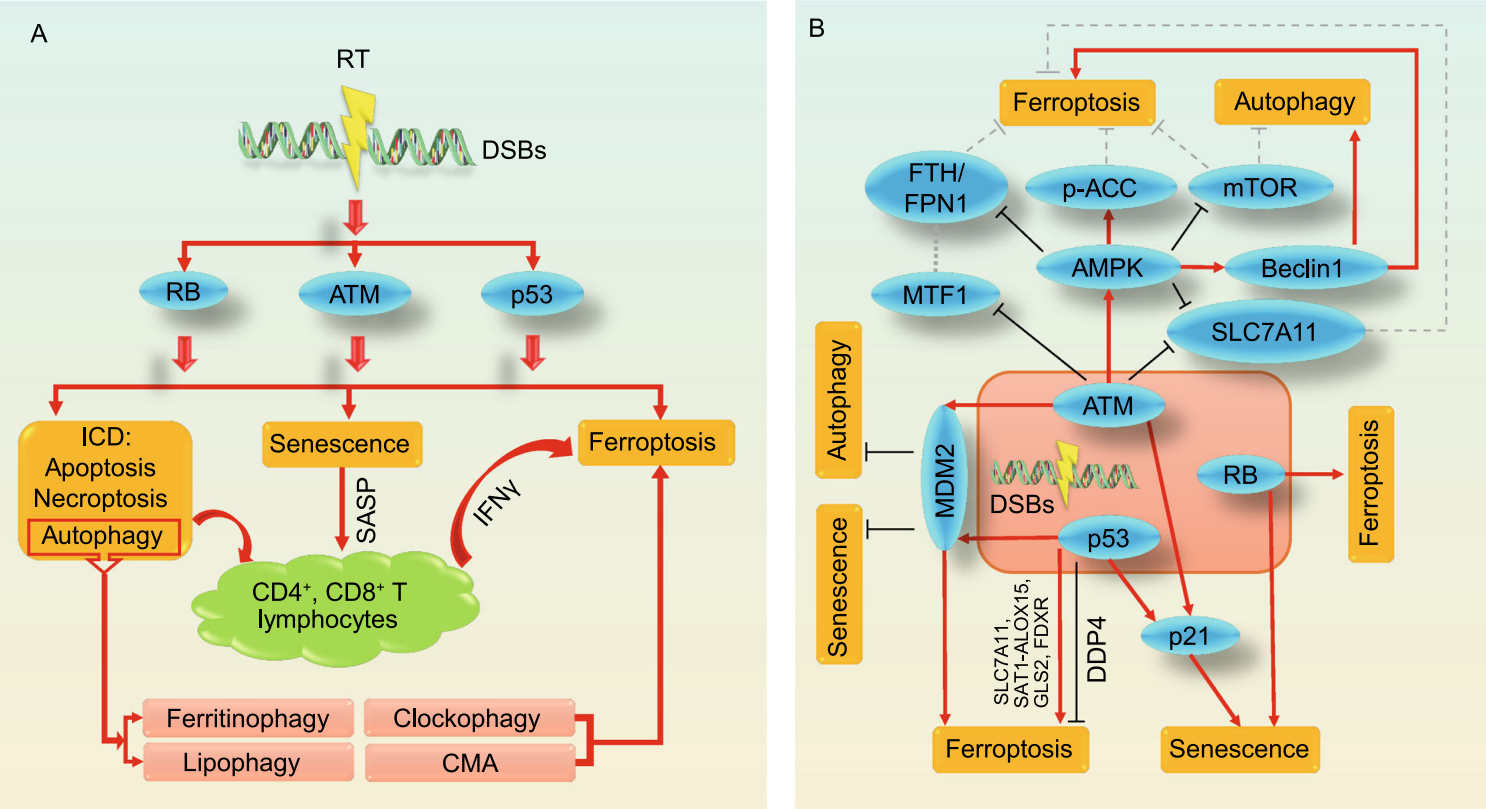
**The crosstalk among RT-induced DSBs, immune system activation and ferroptosis.** (A) Radiotherapy (RT) induces DSBs and thus activates ATM, p53 and RB, promoting senescence, apoptosis, necroptosis, autophagy, and ferroptosis. Immunogenic cell deaths (ICDs; including apoptosis, necroptosis, and autophagy), together with RT-induced senescence-associated secretory phenotype (SASP), contribute to T cell activation, which secretes IFNγ to further promote RT-induced ferroptosis. Additionally, RT-induced autophagy may modulate ferroptosis through ferritinophagy, lipophagy, clockophagy or chaperone-mediated autophagy (CMA). (B) Specific crosstalk mechanisms between ferroptosis and other forms of regulated cell death under RT-induced DSBs, in which ATM, p53 and RB play central roles

However, from a signaling perspective, there seems to exist a crosstalk between DNA damage response and ferroptosis. IR induces DNA damage and thus activates ATM, p53, or RB (Sabin and Anderson, 2011; Maier et al., 2016), which can be linked to RT-induced ferroptosis and other types of RCD, including apoptosis, necroptosis and autophagy, collectively known as immunogenic cell death (ICD) (Kang and Tang, 2016) (Fig. 3A). These ICDs, together with RT-induced senescence-associated secretory phenotype (SASP), activate T cells and recruit them into tumor sites (Rao and Jackson, 2016; Herrera et al., 2017; Li et al., 2018), whereas interferon gamma (IFNγ) secreted from CD8^+^ T cells further promotes RT-induced ferroptosis (Lang et al., 2019; Wang et al., 2019b) (Fig. 3A). Moreover, RT-induced autophagy can potentially promote ferroptosis through ferritinophagy, lipophagy, clockophagy and/or chaperone-mediated autophagy (Liu et al., 2020a) (Fig. 3A). Therefore, the immune system and autophagy may be involved in the intersection between RT-induced DNA damage and ferroptosis.

On the molecular level, multiple regulators possibly underlie the ATM- or p53-mediated crosstalk between ferroptosis and other types of RCD upon IR-induced DSBs (Fig. 3B). IR-induced activation of ATM mediates the downregulation of SLC7A11 expression, thereby contributing to IR-induced ferroptosis (Lang et al., 2019); ATM activation can also promote ferroptosis through the metal regulatory transcription factor 1 (MTF1)-Ferritin/FPN1 axis (Chen et al., 2020) (Fig. 3B). p53 can also be linked to the crosstalk between senescence and ferroptosis. As discussed earlier, p53 transcriptional target p21 suppresses ferroptosis by maintaining GSH levels (Tarangelo et al., 2018); p21 also promotes senescence in cells with irreparable DNA damage (Georgakilas et al., 2017) (Fig. 3B). AMPK is activated in an ATM-dependent manner to mediate multiple cellular effects upon IR-induced DSBs (Sanli et al., 2014). Besides regulating p21-mediated senescence, AMPK is at least partially responsible for IR-induced autophagy by rescinding mammalian target of rapamycin (mTOR)-mediated autophagy inhibition or promoting beclin1-mediated autophagy (Sanli et al., 2014; Zhang et al., 2016); on the other hand, mTOR, beclin1, and AMPK-mediated ACC phosphorylation can modulate ferroprosis, positively or negatively (Song et al., 2018; Lee et al., 2020; Yi et al., 2020) (Fig. 3B). In addition, MDM2 can be up-regulated by IR in an ATM- or p53-dependent fashion (Chen et al., 1994; Maya et al., 2001); subsequently, MDM2 promotes ferroptosis (Venkatesh et al., 2020), but suppresses autophagy and senescence (Wu and Prives, 2018; Liu et al., 2019a; Liu et al., 2020b) (Fig. 3B). Another crosstalk between senescence and ferroptosis is the RB protein, which mediates cellular senescence upon IR and also potentiates ferroptosis (Sabin and Anderson, 2011; Louandre et al., 2015) (Fig. 3B).

In summary, multiple lines of evidence suggest that RT-induced ferroptosis does not affect DNA damage, whereas RT-induced DNA damage appears to affect ferroptosis through diverse mechanisms. There also exist multiple layers of crosstalk between ferroptosis and other RT-induced cellular effects. Further characterization of these interactions may yield new insights into the mechanisms of radioresistance and strategies for radiosensitization, which will be further discussed in the next section.

## THERAPEUTIC POTENTIAL OF FERROPTOSIS IN RT-MEDIATED TUMOR SUPPRESSION

RT destroys tumors precisely in localized areas by IR with a high objective response rate (ORR) and activates the immune system to attack target lesions and distant metastases by inducing ICD (Thariat et al., 2013; Herrera et al., 2017). However, intrinsic or acquired radioresistance is a long-standing challenge in RT; as such, RT is generally combined with other therapies, including chemotherapy, targeted therapy, and immunotherapy, to improve the radiosensitivity and to eliminate potential cancer cells outside the radiation field. In view of this, the landing points for investigating the therapeutic relevance of ferroptosis in RT include: 1) whether ferroptosis and its regulators modulate radiosensitivity, 2) whether targeting ferroptosis contributes to radiosensitization, and 3) how to further incorporate immunotherapy into targeting ferroptosis in RT. Our following discussion in this section will center on these questions.

### Ferroptosis-mediated radiosensitization

**Figure 4:**
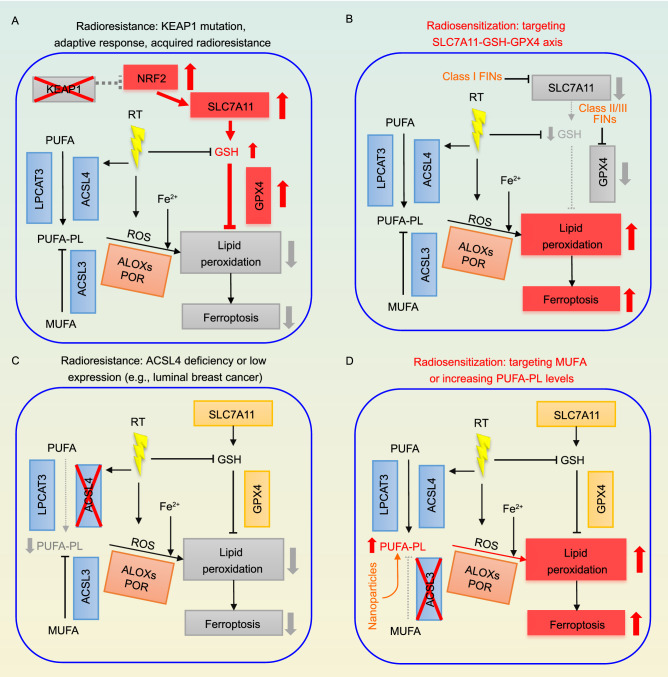
**Radioresistance mechanisms due to ferroptosis inactivation and radiosensitization strategies by inducing ferroptosis.** (A) Radiotherapy (RT) induces the expression of SLC7A11 or GPX4 as an adaptive response to protect cancer cells from ferroptosis, thereby compromising RT-induced cell death and possibly contributing to acquire radioresistance. In addition, *KEAP1* mutant cancer cells are radioresistant due to ferroptosis resistance partly caused by high SLC7A11 expression. (B) Class I FINs targeting SLC7A11 or class II/III FINs targeting GPX4 potentiate RT-induced lipid peroxidation and ferroptosis, thereby sensitizing cancer cells to RT. (C) ACSL4 deficiency or low expression (such as in luminal breast cancer) inhibits RT-induced ferroptosis by blocking PUFA-PL synthesis, resulting in radioresistance. (D) Inhibiting ACSL3-mediated MUFA-PL synthesis or increasing PUFA-PL levels via nanoparticles promotes RT-induced ferroptosis, thereby sensitizing cancer cells to RT

Pharmacological blockade of ferroptosis was shown to protect cancer cells from RT, and RT induced less potent lipid peroxidation and ferroptosis in FIN-resistant cancer cells, which also appear to be radioresistant (Lang et al., 2019; Lei et al., 2020; Ye et al., 2020). Several studies uncovered that IR induces the expression of SLC7A11 and GPX4 as an adaptive response to protect cells from ferroptosis, contributing to radioresistance (Fig. 4A); consequently, depletion or inhibition of SLC7A11 (or GPX4) enables significant radiosensitization by boosting IR-induced ferroptosis (Xie et al., 2011; Pan et al., 2019; Lei et al., 2020) (Fig. 4B). Likewise, deficiency of the tumor suppressor *KEAP1* (which is frequently mutated in lung cancer) inhibited IR-induced ferroptosis at least partly through stabilizing NRF2 and upregulating SLC7A11, leading to radioresistance (Lei et al., 2020) (Fig. 4A). Further, inactivation of ACSL4 impaired the biosynthesis of PUFA-PLs, thereby inhibiting IR-induced ferroptosis and causing radioresistance (Lang et al., 2019; Lei et al., 2020) (Fig. 4C), whereas ablation of ACSL3 diminishes the biosynthesis of MUFA-PLs (which suppress ferroptosis (Magtanong et al., 2019)), leading to enhanced IR-induced ferroptosis and radiosensitization in cancer cells (Lang et al., 2019) (Fig. 4D). Whether other ferroptosis regulators may also modulate radiosensitivity requires further investigation.

Many tumors exhibit at least somewhat susceptibilities to ferroptosis, and tumor sensitivities to RT among different tumor types do not appear to strictly correlate with their susceptibilities to ferroptosis. For example, tumor types in which RT is an important treatment modality, such as hepatocellular carcinoma, pancreatic cancer, diffuse large B cell lymphoma, and triple-negative breast cancer, as well as radioresistant tumor types, including renal cell carcinoma and ovarian cancer, are all somewhat sensitive to ferroptosis (Zou and Schreiber, 2020). This susceptibility may be associated with the high dependence of some tumors, such as a portion of pancreatic cancer and renal cell carcinoma, on cystine uptake. Intriguingly, certain treatment-resistant cancer cells, such as therapy-resistant mesenchymal cancer cells or drug-tolerant persister cancer cells, are vulnerable to ferroptosis, likely because certain unique state of these cancer cells somehow renders them to be particularly dependent on GPX4 function (Hangauer et al., 2017; Viswanathan et al., 2017); likewise, melanoma cells undergoing dedifferentiation upon BRAF inhibitor treatment also exhibit an increased susceptibility to ferroptosis (Tsoi et al., 2018). It was further shown that withaferin A-induced ferroptosis suppresses tumor growth and recurrence in therapy-resistant high-risk neuroblastoma (Hassannia et al., 2018). Further investigations of tumor susceptibility to ferroptosis will help guide strategies to augment RT in cancer treatment.

**Figure 5:**
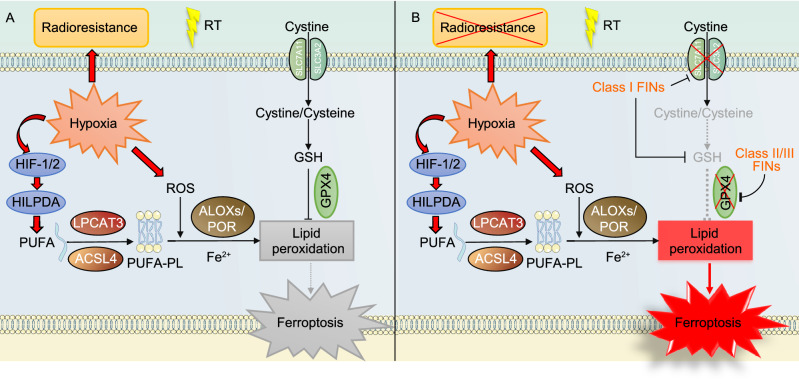
**Interactions of hypoxia with ferroptosis and radioresistance, and potential strategies targeting ferroptosis to overcome hypoxia-induced radioresistance.** (A) Hypoxia causes radioresistance possibly through “oxygen fixation hypothesis” and HIF activation. Hypoxia also induces the levels of ROS and HIF1/2, which have been shown to promote lipid peroxidation. Therefore, this regulation can potentially increase the susceptibility of cancer cells to ferroptosis. However, hypoxia might also upregulate ferroptosis defense systems (e.g., the SLC7A11-GSH-GPX4 axis) to counteract ferroptosis. (B) By inhibiting ferroptosis defense systems, FINs promote RT-induced ferroptosis and might overcome the radioresistance caused by hypoxia

The hypoxic tumor microenvironment represents an important mechanism of radioresistance (Fig. 5A), which is likely attributed to the “oxygen fixation hypothesis” and hypoxia-inducible factors (HIFs) activation (Wang et al., 2019a). On the other hand, hypoxia promotes ROS production (Fig. 5A); consequently, hypoxic tumor cells strongly rely on antioxidant systems to maintain redox homeostasis, and GSH inhibition was shown to overcome hypoxia-mediated radioresistance (Bump and Brown, 1990; Wang et al., 2019a). As discussed above, ROS contributes to POR-mediated lipid peroxidation and ferroptosis(Yan et al., 2020) (Fig. 5A). Intriguingly, HIFs (HIF-1 and -2) have been reported to confer susceptibility to ferroptosis (Fig. 5A). Mechanistically, HIF-2ɑ activates hypoxia-induced, lipid droplet-associated protein (HILPDA) to promote the formation of PUFA-PLs and thereby increase the susceptibility of cancer cells to ferroptosis (Singhal et al., 2019; Zou et al., 2019). HIF-1ɑ activation also sensitizes renal cancer cells to ferroptosis (Zou et al., 2019). In brief summary, while it is well established that hypoxia promotes radioresistance, hypoxia-induced ROS and HIF activation appear to promote ferroptosis (Fig. 5A). More studies are required to clarify the likelihood and specific mechanisms by which ferroptosis induction can reduce radioresistance of hypoxic cancer cells in different cancer settings (Fig. 5B).

In contrast, some drug-resistance cancers that may only respond to RT treatment develop the ability to evade ferroptosis (Boumahdi and de Sauvage, 2020). For example, luminal breast cancer is generally associated with low expression of ACSL4 (Doll et al., 2017), and some breast cancer cells upregulate prominin2 expression to promote iron excretion in the form of ferritin, rendering such cells resistant to ferroptosis (Brown et al., 2019). *KRAS* mutant lung cancers frequently overexpress ACSL3 and thus are equipped to synthesize more MUFA-PLs to protect against ferroptosis (Padanad et al., 2016), while a portion of lung adenocarcinoma cells exhibit high expression of iron-sulfur cluster biosynthesis enzyme cysteine desulfurase (NFS1), thus limiting the reactive iron available for ferroptosis by storing iron in iron-responsive proteins (Alvarez et al., 2017). In addition, anaplastic lymphoma kinase (ALK) positive lymphoma cells can shift the cholesterol synthesis to squalene formation, thereby counteracting lipid peroxidation and ferroptosis (Garcia-Bermudez et al., 2019). A common barrier for cancer therapy is the NRF2 activation in response to therapeutic stress (de la Vega et al., 2018), which also serves as a potential crosstalk between radioresistance and ferroptosis resistance. Overall, ferroptosis plays an important role in RT-mediated tumor suppression, and therefore inducing ferroptosis in RT-resistant tumors represents a promising strategy for radiosensitization. However, for ferroptosis-resistant tumors, which are also likely to be radioresistant, how to minimize their dual resistance remains to be further studied.

### Combining RT with FINs for tumor radiosensitization

As discussed above, genetic perturbation of the anti-ferroptosis systems promotes radiosensitization in diverse cancer cells. Preclinical analyses by several recent studies also showed that FINs can synergize with RT in cancer treatment (Lang et al., 2019; Lei et al., 2020; Ye et al., 2020). For example, class I FINs targeting SLC7A11, such as erastin and sulfasalazine (SAS), class II FINs targeting GPX4, such as RSL3 and ML162, and class III FINs depleting CoQ and GPX4, such as FIN56, could all sensitize non-small cell lung cancer cells to RT *in vitro* (Lei et al., 2020) (Fig. 4B). Further, SAS (an FDA approved drug that is capable of inhibiting SLC7A11) was shown to exhibit a significant radiosensitizing effect in both cell line-derived xenografts (CDXs) and patient-derived xenografts (PDXs) of ovarian cancer and *KEAP1* mutant lung cancer; importantly, ferroptosis inhibitor treatment confirmed that SAS-mediated radiosensitization was indeed mediated by ferroptosis induction (Lang et al., 2019; Lei et al., 2020). Likewise, it was demonstrated that cyst(e)inase (which degrades extracellular cystine and cysteine and therefore operates similar to class I FINs), could sensitize cancer cells or tumors to RT (Lang et al., 2019). Another study showed that RT in combination with imidazole ketone erastin (IKE) or sorafenib (both of which are class I FINs inhibiting SLC7A11 activity) caused dramatic tumor suppression in both CDXs and PDXs (Ye et al., 2020) (Fig. 4B). In all of these studies, the combination of class I FINs with RT appeared to be well tolerated *in vivo*. Collectively, these studies suggest that administration of compounds targeting SLC7A11 to promote RT-induced ferroptosis is likely a promising strategy for radiosensitization *in vivo*, which is in line with other studies targeting SLC7A11 for tumor suppression (Badgley et al., 2020; Hu et al., 2020). Moreover, *Slc7a11* deficient mice are viable with no overt phenotype (Sato et al., 2005; McCullagh and Featherstone, 2014), further indicating the safety of SLC7A11 inhibitors.

Inhibiting GSH synthesis or targeting GPX4 could provide an alternative approach for radiosensitization, especially considering that certain drug-resistant cancer cells are highly dependent on GPX4 for survival (Hangauer et al., 2017) (Fig. 4B). Inhibition of GSH synthesis by buthionine sulphoximine (BSO) to sensitize cancer cells to RT has been well established (Bump and Brown, 1990). Recent studies also showed that RSL3, ML162, and FIN56 have potent radiosensitizing effects *in vitro* (Lang et al., 2019; Lei et al., 2020; Ye et al., 2020); however, these drugs are not suitable for *in vivo* treatment due to their suboptimal pharmacokinetics (Hangauer et al., 2017). In this regard, withaferin A and altretamine (FDA-approved drugs for cancer therapy), with function to inhibit GPX4, exhibited favorable anti-tumor activity in animal models, representing another option for targeting GPX4 *in vivo* (Woo et al., 2015; Hassannia et al., 2018). It should be noted that *Gpx4* knockout mice are embryonic lethal, thereby raising concerns on potential toxicity issues of GPX4 inhibitors for *in vivo* treatment (Yoo et al., 2012; Angeli et al., 2014); however, some cancers appear to be more sensitive to GPX4 inhibitors compared to their corresponding normal cells (Zou et al., 2019), suggesting that there might exist a therapeutic window for targeting GPX4 in certain cancers. Further studies are required to define the therapeutic window of GPX4 inhibition in cancer treatment and to explore techniques to target GPX4 locally in tumors for radiosensitization.

### The relevance of ferroptosis to RT combined with immunotherapy

Immune system activation is an integral part of RT-mediated anticancer effects. On one hand, RT induces ICDs to expose tumor antigens and to activate antigen presenting cells (e.g., dendritic cells), promoting the migration of dendritic cells to the draining lymph nodes, leading to T cell initiation in the lymph nodes and subsequent infiltration of CD8^+^ T cells into the irradiated field or unirradiated distant tumor sites. On the other hand, RT reprograms the tumor microenvironment to favor the recruitment and functioning of effector T cells, making tumor cells more susceptible to T cell attack (Herrera et al., 2017). However, RT also upregulates the expression of programmed death-ligand 1 (PD-L1), a major checkpoint protein in the tumor immunosuppressive microenvironment, which assists cancer cells to escape from T-cell attack (Kordbacheh et al., 2018).

Notably, recent studies identified ferroptosis as a novel intersection between immunotherapy and RT (Lang et al., 2019; Wang et al., 2019b). It was shown that activated CD8^+^ T cells secrete IFNγ during immunotherapy, which down-regulates the expression of SLC7A11 (as well as its regulatory partner SLC3A2) and subsequently inhibits cystine uptake in cancer cells, thereby augmenting lipid peroxidation and ferroptosis. The combination of immune checkpoint inhibitors (ICIs) with cyst(e)inase potentiated tumor ferroptosis and T cell-mediated antitumor immune responses *in vivo*. SLC7A11 expression in tumors was found to negatively correlate with CD8^+^ T cell counts and IFNγ expression in tumors, and prognosis of cancer patients (Wang et al., 2019b). Further, IFNγ secreted by CD8^+^ T cells was shown to promote RT-induced ferroptosis, which is likely caused by the synergistic repression of SLC7A11 expression by RT and IFNγ. ICIs, including PD-L1 or cytotoxic T-lymphocyte-associated protein 4 (CTLA-4) antibodies, in combination with RT synergistically induced tumor ferroptosis, while blocking ferroptosis, pharmacologically or genetically, attenuated the therapeutic effectiveness afforded by combining ICIs with RT; conversely, the therapeutic synergy of immunotherapy and RT could be further enhanced by inactivating SLC7A11 in tumors (Lang et al., 2019).

RT is commonly administered in combination with immunotherapy, particularly ICIs, but this combination therapy seem to lack the expected survival benefits in certain tumors (Malhotra et al., 2017), highlighting an urgent need to identify specific biomarkers to define which tumors could be sensitive to the combination therapy. In this regard, tumors with low expression of anti-ferroptosis genes (such as SLC7A11) and/or high expression of pro-ferroptosis genes (such as ACSL4) could indicate that such tumors are particularly susceptible to ferroptosis and therefore might be suitable for this combination therapy. For those tumors that exhibit ferroptosis resistance features (such as with high expression of anti-ferroptosis genes and/or low expression of pro-ferroptosis genes), combining FINs with immunotherapy and RT might be a good strategy to enhance tumor ferroptosis and sensitize such tumors to immunotherapy and RT; however, whether this triple therapy strategy will also increase toxicity in normal tissues remains to be determined.

## CONCLUSIONS AND FUTURE PERSPECTIVES

Recent studies establish a critical role of ferroptosis in RT and further suggest therapeutic strategies to target ferroptosis in RT as well as immunotherapy. Below we highlight a few key questions for further translating these findings into clinical applications. First, there exist significant differences in radiosensitivity among different types of cancer; even within the same tumor type, radiosensitivity might vary considerably among individuals due to tumor heterogeneity. Therefore, identifying suitable biomarkers for individualized RT has been an unmet need in RT research. In this regard, elevated levels of ferroptosis marker 4-HNE were found in tumor samples from patients treated with RT compared with matched tumor samples before RT; importantly, patients with strongly-positive levels of 4-HNE appeared to have better RT response and longer survival than those with weak/moderate levels of 4-HNE, suggesting an important role of ferroptosis in patients receiving RT (Lei et al., 2020). Therefore, further dissecting the interaction between RT and ferroptosis and using this information to develop mechanism-based biomarkers for patient stratification may help identify radiosensitive individuals and define populations suitable for co-treatment with FINs.

Further, although FIN + RT combination therapies appeared to be safe in preclinical studies, other studies demonstrated that ferroptosis might also be involved in RT-induced normal tissue damage, such as RT-induced lung injury (Li et al., 2019a; Li et al., 2019b). Therefore, it will be important to further clarify whether RT combination with FIN causes less toxicities to normal tissues than tumors (i.e., whether there exists an optimal therapeutic window). The development of nanomaterials with FIN activity may be an alternative way to address this issue.

Finally, current studies investigating the role of ferroptosis in RT have focused on X-rays, a type of photon with low LET. With the continuous development of RT physics, proton therapy and other high LET radiations, such as carbon ions, have been developed in recent years, and some of them are shown to achieve superior therapeutic efficacies than photon therapy (Mohan and Grosshans, 2017; Mohamad et al., 2019). Correspondingly, it will be interesting to explore the potential role of ferroptosis in proton and other high LET radiations.

Together, a comprehensive understanding of these points will allow for further clarification of the mechanisms underlying IR-induced ferroptosis and for more robust establishment of therapeutic strategies targeting ferroptosis in RT, offering opportunities to develop superior FINs for radiosensitization.
